# Bis(3-hydroxy­phenyl­acetato-κ^2^ *O*,*O*′)bis­(1*H*-imidazole-κ*N* ^3^)nickel(II)

**DOI:** 10.1107/S1600536809005960

**Published:** 2009-02-28

**Authors:** Xiao-Yan Nie, Qian-Zhu Li

**Affiliations:** a Department of Chemistry, Bijie University, Bijie 551700, People’s Republic of China

## Abstract

In the title mononuclear complex, [Ni(C_8_H_7_O_3_)_2_(C_3_H_4_N_2_)_2_], the Ni^II^ atom, lying on a twofold rotation axis, is coordinated by four carboxyl­ate O atoms from two bidentate 3-hydroxy­phenylacetato ligands and two N atoms from two imidazole mol­ecules in a distorted octa­hedral geometry. A three-dimensional network is formed *via* inter­molecular O—H⋯O and N—H⋯O hydrogen bonds and π–π stacking inter­actions between the imidazole and benzene rings of neighboring mol­ecules [centroid–centroid distance = 3.856 (2) Å].

## Related literature

For the use of coordination polymers and open framework materials in catalysis, separation, gas storage and molecular recognition, see: James (2003 ▶); Serre *et al.* (2004 ▶); Yaghi *et al.* (1998 ▶, 2003 ▶).
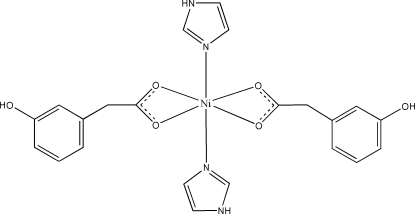


## Experimental

### 

#### Crystal data

[Ni(C_8_H_7_O_3_)_2_(C_3_H_4_N_2_)_2_]
*M* *_r_* = 497.15
Monoclinic, 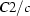

*a* = 12.8481 (14) Å
*b* = 10.6829 (12) Å
*c* = 16.3051 (19) Å
β = 101.410 (1)°
*V* = 2193.7 (4) Å^3^
*Z* = 4
Mo *K*α radiation
μ = 0.93 mm^−1^
*T* = 296 K
0.32 × 0.27 × 0.24 mm

#### Data collection

Bruker APEXII CCD diffractometer
Absorption correction: multi-scan (*SADABS*; Sheldrick, 1996 ▶) *T*_min_ = 0.762, *T*_max_ = 0.811
5522 measured reflections
1960 independent reflections
1509 reflections with *I* > 2σ(*I*)
*R*_int_ = 0.045

#### Refinement

*R*[*F* ^2^ > 2σ(*F* ^2^)] = 0.039
*wR*(*F* ^2^) = 0.094
*S* = 1.01
1960 reflections
151 parameters
H-atom parameters constrained
Δρ_max_ = 0.29 e Å^−3^
Δρ_min_ = −0.25 e Å^−3^

### 

Data collection: *APEX2* (Bruker, 2007 ▶); cell refinement: *SAINT* (Bruker, 2007 ▶); data reduction: *SAINT*; program(s) used to solve structure: *SHELXS97* (Sheldrick, 2008 ▶); program(s) used to refine structure: *SHELXL97* (Sheldrick, 2008 ▶); molecular graphics: *SHELXTL* (Sheldrick, 2008 ▶); software used to prepare material for publication: *SHELXTL*.

## Supplementary Material

Crystal structure: contains datablocks I, global. DOI: 10.1107/S1600536809005960/hy2181sup1.cif

Structure factors: contains datablocks I. DOI: 10.1107/S1600536809005960/hy2181Isup2.hkl

Additional supplementary materials:  crystallographic information; 3D view; checkCIF report

## Figures and Tables

**Table 1:** Selected bond lengths (Å)

Ni1—N1	2.004 (2)
Ni1—O2	2.1128 (19)
Ni1—O1	2.1404 (18)

**Table 2:** Hydrogen-bond geometry (Å, °)

*D*—H⋯*A*	*D*—H	H⋯*A*	*D*⋯*A*	*D*—H⋯*A*
N2—H2*A*⋯O1^i^	0.86	1.88	2.734 (3)	171
O3—H3⋯O2^ii^	0.82	1.96	2.763 (3)	168

Symmetry codes: (i) 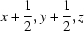; (ii) 
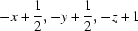
.

**Fig. 1.**
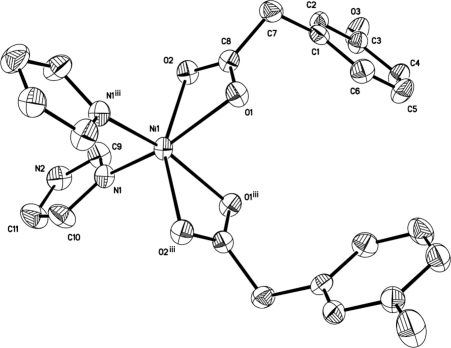
The molecular structure of the title compound. Displacement ellipsoids are drawn at the 30% probability level. [Symmetry code: (iii) -x, y, 1/2 - z.]

**Fig. 2.**
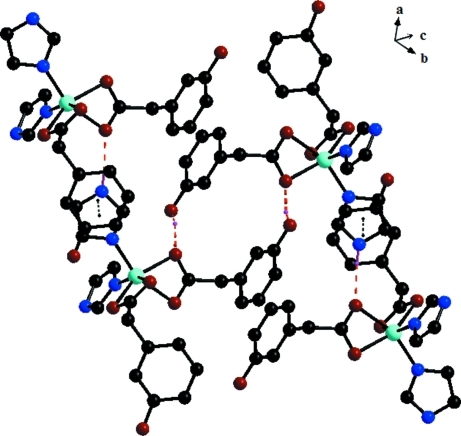
A packing view of the title compound. The intermolecluar hydrogen bonds and π–π stacking interactions are shown as dashed lines.

## supplementary crystallographic information

### Comment

The use of multifunctional organic linker molecules in the preparation of coordination polymers and open-framework materials has led to the development of a rich field of chemistry, owing to the potential applications of these materials in catalysis, separation, gas storage and molecular recognition (James, 2003; Serre *et al.*, 2004; Yaghi *et al.*, 1998, 2003). 3-Hydroxyphenylacetic acid has a versatile binding ability. Structures of the complexes with 3-hydroxyphenylacetate have not been reported to date. We obtained a new Ni^II^ complex from the reaction of 3-hydroxyphenylacetic acid, imidazole and NiCl_2_ in an alkaline aqueous solution.

As illustrated in Fig. 1, the Ni^II^ atom, lying on a twofold rotation axis, has a distorted octahedral environment, defined by four carboxylate O atoms from two bidentate 3-hydroxyphenylacetate ligands and two N atoms from two imidazole molecules. Intermolecular O—H···O and N—H···O hydrogen bonds form a three-dimensional network, which is further consolidated by π–π stacking interactions between the imidazole and phenyl rings of neighboring complexes, with a centroid–centroid distance of 3.856 (2) Å.

### Experimental

A mixture of nickel chloride (0.13 g, 1 mmol), 3-hydroxyphenylacetic acid (0.152 g, 1 mmol), imidazole (0.068 g, 1 mmol), NaOH (0.06 g, 1.5 mmol) and H_2_O (12 ml) was placed in a 23 ml Teflon-lined reactor, which was heated to 433 K for 3 d and then cooled to room temperature at a rate of 10 K h^-1^. The crystals obtained were washed with water and dried in air.

### Refinement

H atoms were positioned geometrically and treated as riding on the parent C atoms, with C—H = 0.93 (CH) and 0.97 (CH_2_) Å, N—H = 0.86 Å, and with *U*_iso_(H) = 1.2*U*_eq_(C, N).

### Crystal data

**Table:**

[Ni(C_8_H_7_O_3_)_2_(C_3_H_4_N_2_)_2_]	*F*(000) = 1032
*M**_r_* = 497.15	*D*_x_ = 1.505 Mg m^−^^3^
Monoclinic, *C*2/*c*	Mo *K*α radiation, λ = 0.71073 Å
Hall symbol: -C 2yc	Cell parameters from 5837 reflections
*a* = 12.8481 (14) Å	θ = 2.8–27.9°
*b* = 10.6829 (12) Å	µ = 0.93 mm^−^^1^
*c* = 16.3051 (19) Å	*T* = 296 K
β = 101.410 (1)°	Block, blue
*V* = 2193.7 (4) Å^3^	0.32 × 0.27 × 0.24 mm
*Z* = 4	

### Data collection

**Table:**

Bruker APEXII CCD diffractometer	1960 independent reflections
Radiation source: fine-focus sealed tube	1509 reflections with *I* > 2σ(*I*)
graphite	*R*_int_ = 0.045
φ and ω scans	θ_max_ = 25.2°, θ_min_ = 2.5°
Absorption correction: multi-scan (*SADABS*; Sheldrick, 1996)	*h* = −13→15
*T*_min_ = 0.762, *T*_max_ = 0.811	*k* = −10→12
5522 measured reflections	*l* = −19→19

### Refinement

**Table:**

Refinement on *F*^2^	Primary atom site location: structure-invariant direct methods
Least-squares matrix: full	Secondary atom site location: difference Fourier map
*R*[*F*^2^ > 2σ(*F*^2^)] = 0.039	Hydrogen site location: inferred from neighbouring sites
*wR*(*F*^2^) = 0.094	H-atom parameters constrained
*S* = 1.01	*w* = 1/[σ^2^(*F*_o_^2^) + (0.0443*P*)^2^] where *P* = (*F*_o_^2^ + 2*F*_c_^2^)/3
1960 reflections	(Δ/σ)_max_ < 0.001
151 parameters	Δρ_max_ = 0.29 e Å^−^^3^
0 restraints	Δρ_min_ = −0.25 e Å^−^^3^

### Fractional atomic coordinates and isotropic or equivalent isotropic displacement parameters (Å^2^)

**Table:**

	*x*	*y*	*z*	*U*_iso_*/*U*_eq_	
C1	0.0902 (2)	0.0767 (3)	0.43721 (17)	0.0387 (7)	
N1	0.10445 (18)	0.5333 (2)	0.22611 (15)	0.0392 (6)	
Ni1	0.0000	0.40576 (5)	0.2500	0.03283 (19)	
O1	−0.06407 (15)	0.25890 (17)	0.31434 (12)	0.0421 (5)	
C2	0.1936 (2)	0.0654 (3)	0.48209 (18)	0.0438 (8)	
H2	0.2208	0.1263	0.5213	0.053*	
N2	0.2479 (2)	0.6473 (3)	0.24415 (17)	0.0543 (8)	
H2A	0.3104	0.6747	0.2651	0.065*	
O2	0.07730 (15)	0.36060 (19)	0.37318 (12)	0.0423 (5)	
C3	0.2567 (2)	−0.0347 (3)	0.46956 (19)	0.0451 (8)	
O3	0.35853 (19)	−0.0487 (2)	0.51162 (17)	0.0704 (8)	
H3	0.3769	0.0141	0.5397	0.106*	
C4	0.2166 (3)	−0.1259 (3)	0.4120 (2)	0.0521 (9)	
H4	0.2580	−0.1945	0.4040	0.063*	
C5	0.1146 (3)	−0.1143 (3)	0.3666 (2)	0.0566 (9)	
H5	0.0877	−0.1751	0.3272	0.068*	
C6	0.0517 (3)	−0.0144 (3)	0.37856 (19)	0.0503 (8)	
H6	−0.0169	−0.0079	0.3472	0.060*	
C7	0.0230 (3)	0.1885 (3)	0.45013 (18)	0.0476 (8)	
H7A	−0.0462	0.1602	0.4578	0.057*	
H7B	0.0565	0.2332	0.5002	0.057*	
C8	0.0100 (2)	0.2748 (3)	0.37584 (17)	0.0369 (7)	
C9	0.1992 (2)	0.5532 (3)	0.2722 (2)	0.0534 (9)	
H9	0.2283	0.5060	0.3191	0.064*	
C10	0.0930 (3)	0.6207 (3)	0.1660 (2)	0.0586 (10)	
H10	0.0335	0.6304	0.1236	0.070*	
C11	0.1815 (3)	0.6928 (4)	0.1766 (2)	0.0699 (11)	
H11	0.1939	0.7602	0.1437	0.084*	

### Atomic displacement parameters (Å^2^)

**Table:**

	*U*^11^	*U*^22^	*U*^33^	*U*^12^	*U*^13^	*U*^23^
C1	0.0439 (19)	0.0382 (18)	0.0339 (16)	0.0014 (14)	0.0076 (14)	0.0093 (14)
N1	0.0313 (14)	0.0370 (14)	0.0487 (16)	−0.0043 (11)	0.0066 (12)	−0.0007 (12)
Ni1	0.0233 (3)	0.0304 (3)	0.0417 (3)	0.000	−0.0010 (2)	0.000
O1	0.0325 (11)	0.0430 (12)	0.0454 (12)	−0.0088 (9)	−0.0050 (10)	0.0045 (10)
C2	0.055 (2)	0.0348 (18)	0.0385 (17)	−0.0029 (14)	0.0013 (16)	−0.0036 (13)
N2	0.0370 (16)	0.0630 (19)	0.0620 (19)	−0.0242 (14)	0.0077 (14)	−0.0082 (15)
O2	0.0316 (11)	0.0438 (12)	0.0458 (12)	−0.0053 (10)	−0.0060 (10)	−0.0007 (10)
C3	0.0437 (19)	0.0416 (18)	0.0447 (18)	0.0013 (15)	−0.0042 (15)	−0.0026 (15)
O3	0.0543 (15)	0.0611 (16)	0.0821 (19)	0.0126 (13)	−0.0195 (14)	−0.0175 (14)
C4	0.054 (2)	0.0443 (19)	0.053 (2)	0.0052 (16)	0.0004 (18)	−0.0078 (16)
C5	0.062 (2)	0.050 (2)	0.054 (2)	−0.0066 (17)	0.0004 (18)	−0.0176 (16)
C6	0.0437 (19)	0.056 (2)	0.047 (2)	−0.0042 (16)	−0.0015 (16)	−0.0030 (17)
C7	0.053 (2)	0.050 (2)	0.0401 (18)	0.0095 (16)	0.0090 (16)	0.0049 (15)
C8	0.0316 (17)	0.0367 (17)	0.0404 (17)	0.0035 (13)	0.0023 (14)	−0.0021 (13)
C9	0.0348 (18)	0.054 (2)	0.066 (2)	−0.0085 (16)	−0.0027 (17)	0.0087 (18)
C10	0.054 (2)	0.069 (3)	0.046 (2)	−0.0209 (18)	−0.0041 (17)	0.0133 (18)
C11	0.080 (3)	0.070 (3)	0.057 (2)	−0.036 (2)	0.006 (2)	0.010 (2)

### Geometric parameters (Å, °)

**Table:**

C1—C6	1.385 (4)	O2—C8	1.267 (3)
C1—C2	1.389 (4)	C3—O3	1.360 (3)
C1—C7	1.513 (4)	C3—C4	1.380 (4)
N1—C9	1.315 (4)	O3—H3	0.8200
N1—C10	1.340 (4)	C4—C5	1.378 (4)
Ni1—N1	2.004 (2)	C4—H4	0.9300
Ni1—N1^i^	2.004 (2)	C5—C6	1.376 (4)
Ni1—O2^i^	2.1128 (19)	C5—H5	0.9300
Ni1—O2	2.1128 (19)	C6—H6	0.9300
Ni1—O1^i^	2.1404 (18)	C7—C8	1.505 (4)
Ni1—O1	2.1404 (18)	C7—H7A	0.9700
O1—C8	1.250 (3)	C7—H7B	0.9700
C2—C3	1.381 (4)	C9—H9	0.9300
C2—H2	0.9300	C10—C11	1.356 (4)
N2—C9	1.313 (4)	C10—H10	0.9300
N2—C11	1.343 (4)	C11—H11	0.9300
N2—H2A	0.8600		
			
C6—C1—C2	118.5 (3)	C11—N2—H2A	126.5
C6—C1—C7	121.0 (3)	C8—O2—Ni1	90.11 (16)
C2—C1—C7	120.5 (3)	O3—C3—C2	123.0 (3)
C9—N1—C10	105.1 (3)	O3—C3—C4	117.2 (3)
C9—N1—Ni1	125.3 (2)	C2—C3—C4	119.8 (3)
C10—N1—Ni1	129.4 (2)	C3—O3—H3	109.5
N1—Ni1—N1^i^	94.31 (13)	C5—C4—C3	119.3 (3)
N1—Ni1—O2^i^	100.22 (9)	C5—C4—H4	120.4
N1^i^—Ni1—O2^i^	97.65 (9)	C3—C4—H4	120.4
N1—Ni1—O2	97.65 (9)	C6—C5—C4	121.1 (3)
N1^i^—Ni1—O2	100.22 (9)	C6—C5—H5	119.4
O2^i^—Ni1—O2	153.60 (11)	C4—C5—H5	119.4
N1—Ni1—O1^i^	93.78 (8)	C5—C6—C1	120.2 (3)
N1^i^—Ni1—O1^i^	158.58 (9)	C5—C6—H6	119.9
O2^i^—Ni1—O1^i^	61.36 (7)	C1—C6—H6	119.9
O2—Ni1—O1^i^	98.31 (8)	C8—C7—C1	110.3 (2)
N1—Ni1—O1	158.58 (9)	C8—C7—H7A	109.6
N1^i^—Ni1—O1	93.78 (8)	C1—C7—H7A	109.6
O2^i^—Ni1—O1	98.31 (8)	C8—C7—H7B	109.6
O2—Ni1—O1	61.36 (7)	C1—C7—H7B	109.6
O1^i^—Ni1—O1	85.73 (10)	H7A—C7—H7B	108.1
N1—Ni1—C8^i^	98.66 (9)	O1—C8—O2	119.2 (3)
N1^i^—Ni1—C8^i^	128.39 (10)	O1—C8—C7	120.5 (3)
O2^i^—Ni1—C8^i^	30.92 (8)	O2—C8—C7	120.2 (3)
O2—Ni1—C8^i^	126.79 (9)	N2—C9—N1	112.0 (3)
O1^i^—Ni1—C8^i^	30.45 (8)	N2—C9—H9	124.0
O1—Ni1—C8^i^	91.74 (8)	N1—C9—H9	124.0
C8—O1—Ni1	89.31 (17)	N1—C10—C11	109.5 (3)
C3—C2—C1	121.2 (3)	N1—C10—H10	125.2
C3—C2—H2	119.4	C11—C10—H10	125.2
C1—C2—H2	119.4	N2—C11—C10	106.2 (3)
C9—N2—C11	107.1 (3)	N2—C11—H11	126.9
C9—N2—H2A	126.5	C10—C11—H11	126.9

Symmetry codes: (i) −*x*, *y*, −*z*+1/2.

### Hydrogen-bond geometry (Å, °)

**Table:**

*D*—H···*A*	*D*—H	H···*A*	*D*···*A*	*D*—H···*A*
N2—H2A···O1^ii^	0.86	1.88	2.734 (3)	171
O3—H3···O2^iii^	0.82	1.96	2.763 (3)	168

Symmetry codes: (ii) *x*+1/2, *y*+1/2, *z*; (iii) −*x*+1/2, −*y*+1/2, −*z*+1.
